# On Non-Random Mating, Adaptive Evolution, and Information Theory

**DOI:** 10.3390/biology13120970

**Published:** 2024-11-25

**Authors:** Antonio Carvajal-Rodríguez

**Affiliations:** Centro de Investigación Mariña (CIM), Departamento de Bioquímica, Genética e Inmunología, Universidade de Vigo, 36310 Vigo, Spain; acraaj@uvigo.es

**Keywords:** non-random mating, population genetics model, Kullback–Leibler divergence, information theory

## Abstract

Population genetics describes evolutionary processes, focusing on the variation within and between species and the forces shaping this diversity. Evolution reflects information accumulated in genomes, enhancing organisms’ adaptation to their environment. In this paper, I propose a model that begins with the distribution of mating based on mutual fitness and progresses to viable adult genotype distribution. At each stage, the changes result in different measures of information. The evolutionary dynamics at each stage of the model correspond to certain aspects of interest, such as the type of mating, the distribution of genotypes in regard to mating, and the distribution of genotypes and haplotypes in the next generation. Changes to these distributions are caused by variations in fitness and result in Jeffrey’s divergence values other than zero. As an example, a model of hybrid sterility is developed of a biallelic locus, comparing the information indices associated with each stage of the evolutionary process. In conclusion, the informational perspective seems to facilitate the connection between cause and effect and allows the development of statistical tests to perform hypothesis testing against zero-information null models (random mating, no selection, etc.). The informational perspective could contribute to clarify, deepen, and expand the mathematical foundations of evolutionary theory.

## Simple Summary

The evolutionary process can be seen as a process of acquisition, storage, and updating of information by a population about the environment in which it lives. In this paper, I propose a model that starts with the distribution of mating that occurs according to mutual mating fitness and ends with the distribution of viable adult genotypes obtained after this mating. The result of the evolutionary dynamics associated with each stage of the model can be described in terms of information. This informational description facilitates the connection between cause and effect, as well as the development of statistics to test the null model of zero information, i.e., random mating and/or no effect of natural selection. Incorporating the informational perspective into the mathematical formalism of population genetics/genomics contributes to clarifying, expanding, and deepening the mathematical description of evolutionary theory.

## 1. Introduction

The evolutionary process involves the accumulation of information in genomes, which allows living beings to improve their adaptation to the environment. We can trace the mathematical treatment of information back to Shannon [1], who, building on previous work by Nyquist [2] and Hartley [3] on communication and information, published his mathematical theory of communication in 1948, which was soon expanded and became useful across a wide range of fields. In biology, there is an abundance of studies proposing different descriptions and uses of information theory in a biological context, see for example [4,5,6]. In population genetics, one of the first authors to consider natural selection as a process of obtaining information about the environment during adaptive evolution was Kimura [7]. Spetner [8] also discussed the transmission of information during adaptive evolution and, later, Maynard Smith [9] and Szathmáry [10] recognized the potential relevance of the concept of information for evolutionary theory. Mac Kay [11] used this formalism to show that recombination allows the acquisition of information due to natural selection at a faster rate than parthenogenesis. In the new century, Adami demonstrated the connection between information theory and the evolution of biological complexity and molecular biology [12,13,14]. In 2001, Frieden et al. [15] posed evolutionary dynamics as a process that maximizes information and Strelioff et al. [16] described the mutual information between loci that captures epistasis. However, it seems to be Frank [17,18,19] who first showed the equivalence between the description in terms of information and the classical dynamic description of population genetics. More recently, other authors have developed and deepened similar ideas [20,21,22].

Focusing on the selective component of the evolutionary process, the classical dynamic description starts with initial frequencies, based on which the force of natural selection acts, allowing us to predict the final frequencies. However, given the significant difficulty in directly measuring the effect of natural selection, the informational approach becomes relevant, where we consider the fluctuation between the frequencies of the ancestral population and the new one, to obtain the information associated with the natural selection process that has caused that fluctuation [17,20]. Thus, we can obtain information about natural selection from the observed frequencies in two separate population groups, either in space or in time.

In summary, population genetics is the formal theory of evolution, focusing on the concepts of natural selection, genetic drift, mutation, and recombination, etc., and the relationship of these concepts with adaptation to the environment and their effect on genetic diversity. However, in recent years, a reformulation of the evolutionary process in terms of information is gaining ground.

This information-based approach has recently been extended to calculate the information gained by comparing the distribution of phenotypes in regard to mating with their distribution in the population. This has enabled the derivation of sexual selection and assortative mating statistics, as well as the design of a hierarchy of models that have enabled the application of multi-model selection techniques to the study of non-random mating [23,24,25].

To develop this description in terms of information, the connection between Price’s equation, which describes the mean phenotypic change due to the effect of selection, and Jeffrey’s divergence was utilized, as established in the work by Frank [18].

In this paper, we propose a model that starts with the distribution of mating that occurs according to mutual mating fitness and ends with the distribution of viable adult genotypes obtained after this mating. The evolutionary dynamics associated with each stage of the model can be described in terms of information.

The structure of the paper is as follows: first, we will briefly review Price’s equation and the notions of entropy and information. Second, we will review recent results related to mating fitness and non-random mating to highlight various connections to some measures and properties of information theory. Third, we propose the inclusion of a mating fitness concept within a more general model that will incorporate the genotype–phenotype map and other elements of the life cycle, such as viability. We will show that the mean change in fitness, caused by the shift in genotypic and haplotypic distributions due to sexual and natural selection, can be expressed as Jeffrey’s divergence. We will explicitly calculate the information for the simple case of a biallelic locus with non-random mating, according to which we will demonstrate the different information measures obtained for a model of hybrid sterility. Finally, we will briefly comment, in light of the above and recent literature, on whether the concept of information acquisition could serve as an alternative to biological fitness as a design principle and the central axis of evolutionary dynamics.

## 2. Price’s Equation

Price’s equation is a mathematical identity that, in its most general form, can be applied to any characteristic that classifies two sets of entities separated in time and/or space [18,19,26,27,28,29]. Furthermore, Price’s equation has been considered to be a fundamental, unifying, and model-generating theorem in evolutionary theory [30,31,32]. Indeed, key theorems of evolution, such as the gene selection theorem (average excess), the fundamental theorem of phenotypic selection (Robertson’s equation), Breeder’s equation, and Fisher’s fundamental theorem, can be derived from it [33], although regarding the latter, see the recent work by Ewens [34].

Price’s equation can be interpreted in terms of what Kuhn [35] called symbolic generalizations or generalization sketches and, as such, it has been connected with many different types of models, based on which new variants of the equation can be derived to solve different types of problems (reviewed in [30]). Furthermore, Price’s equation can also be interpreted in terms of information [18] and, as we have already mentioned, this interpretation has recently been leveraged to generate estimators of sexual selection patterns and assortative mating [23,24,25].

Price’s equation can be formalized in different ways; here, we will follow Frank’s notation to express it as the mean change between two sets of entities for a character, *z*, indexed by different classes, *i*, as follows:

(1) $$\Delta \bar{z}=cov\left(\omega ,z\right)+E\left(\omega ,\Delta z\right)$$

where $\omega =\frac{w}{\bar{w}}.$

## 3. Entropy and Information

Information in this regard is concerned with the decrease in entropy or uncertainty in a system. Shannon [1] defined the uncertainty or entropy of a discrete random variable as *X*, which takes values *x*_i_ with probability *p_i_*, as:

(2) $$H\left(X\right)=-\sum_{i=1}^{N}p_{i}log\left(p_{i}\right)$$

### 3.1. Mutual Information

The maximum uncertainty for a variable with *N* possible states is *H_max_* = log(*N*) [36] and we can define the information, *I*, in a system simply as:

$$I\left(X\right)=H_{max}-H\left(X\right).$$

It is also possible to measure the uncertainty of *X* conditional upon another random variable, *Y*:

(3) $$H\left(X|Y\right)=-\sum_{y}p\left(y\right)\sum_{x}p\left(x|y\right)log\left(p\left(x|y\right)\right)$$

and then the mutual information between *X* and *Y* is the reduction in uncertainty about *X*, thanks to the knowledge we have about:

(4) $$I\left(X;Y\right)=H\left(X\right)-H\left(X|Y\right)$$

### 3.2. Relative Entropy or Kullback–Leibler Divergence

Kullback–Leibler divergence (*D_KL_*) or relative entropy allows us to compare two distributions, *P* and *Q*, and quantifies the information gained by using *P* instead of *Q*.

(5) $$D_{KL}\left(P\left|\right|Q\right)=\sum_{x}P\left(x\right)log\left(\frac{P\left(x\right)}{Q\left(x\right)}\right)$$

It can be shown (the information inequality theorem) that *D_KL_* ≥ 0, the equality being fulfilled only when *P* and *Q* are equal.

The relationship between *D_KL_* and mutual information is:

(6) $$I\left(X;Y\right)=D_{KL}\left(P\left(X,Y\right)\left|\right|P\left(X\right)P\left(Y\right)\right)$$

That is, mutual information quantifies the information gained by using the joint distribution of *X* and *Y*, instead of the product of the two marginal distributions.

We can also relate *D_KL_* to information and maximum entropy, since:

$$I\left(X\right)=H_{max}-H\left(X\right)=D_{KL}\left(X\left|\right|U\right)$$

where *U* is the uniform distribution over *X* [36].

### 3.3. Jeffrey’s Divergency

Jeffrey’s divergence is the symmetrical version of the *D_KL_* divergence, that is, the sum of the two possible divergences between *P* and *Q* and, therefore, it measures the total information that changes when using *Q* instead of *P*, plus that of *P* instead of *Q*:

(7) $$J\left(P,Q\right)=D_{KL}\left(P\left|\right|Q\right)+D_{KL}\left(Q\left|\right|P\right)$$

As a curiosity, *J* is also known as the Population Stability Index in the world of financial risk modeling and analysis.

Both *D_KL_* and *J* divergences are particular cases of the *f* divergence, which is defined for the discrete case as:

$$D_{f}\left(P\left|\right|Q\right)=\sum_{x}Q\left(x\right)f\left(\frac{P\left(x\right)}{Q\left(x\right)}\right)$$

where *f* is the generating function, which for *D_KL_* is *f*(*x*) = *x*log *x* and for *J* is *f*(*x*) = (*x* − 1)log *x*.

## 4. Information-Related Interpretation of Price’s Equation

The first connection between Price’s equation and information metrics was established by Frank [17], connecting Price’s equation with Fisher’s information. The relationship between Price’s equation and relative entropy and Jeffrey’s (*J*) divergence are discussed in later work by the same author [18,19,37,38].

To see the connection with the *J* divergence, let us return to Equation (1), focusing on the first term, which expresses the average change in the character *z* caused by selection [18]:

$$\Delta \bar{z_{s}}=cov\left(\omega ,z\right)=\sum p_{i}\left(z_{i}-\bar{z}\right)\left(\omega_{i}-\bar{\omega }\right)\;\Delta \bar{z_{s}}=\sum p_{i}\left(z_{i}-\bar{z}\right)\left(\omega_{i}-1\right)=\sum p_{i}\left(z_{i}-\bar{z}\right)\left(\frac{p\prime_{i}}{p_{i}}-1\right)=\sum \Delta p_{i}z_{i}$$

Note that the relative fitness is *ω_i_* = *p*’*_i_*/*p_i_*, where *p_i_* is the frequency of class *i* before selection and *p*’*_i_* is the frequency after selection. The average relative fitness is equal to 1, Δ*p_i_* = *p*’*_i_* − *p_i_* and ΣΔ*p_i_* = 0.

If we take as character *z*, the logarithm of the relative fitness, we obtain the expression of the first component of Price’s equation in terms of information [18]:

(8) $$\Delta \bar{z_{s}}=\sum \Delta p_{i}z_{i}=\sum \Delta p_{i}log\left(\omega_{i}\right)=\sum \Delta p_{i}log\left(\frac{p\prime_{i}}{p_{i}}\right)=J\left(P\prime ,P\right)$$

We then see that the mean change in the logarithm of relative fitness is the difference in Jeffrey’s divergence between the frequency distribution after selection (*P*’) and before selection (*P*).

It is interesting to note that:

$$\Delta p=\sum_{i}p\prime_{i}-p_{i}=\sum_{i}\omega_{i}p_{i}-p_{i}=\sum_{i}p_{i}\left(\omega_{i}-1\right)$$

and

$$\Delta \bar{z_{s}}=\sum_{i}\Delta p_{i}z_{i}=\sum_{i}p_{i}\left(\omega_{i}-1\right)log\left(\omega_{i}\right)=\sum_{i}p_{i}f\left(\omega_{i}\right)$$

where *f* is the *f* divergence generating function for *J*, so Equation (8) is an *f* divergence with generating function (*ω −* 1)log *ω*, which explains that the evolution of the distribution caused by the fitness parameter in replicator-type models can always be expressed as Jeffrey’s divergence.

Recently, Carvajal-Rodríguez used these ideas to reinterpret Price’s equation in terms of the comparison between two sets of pairs, one representing random mating (*Q*) and the other (*Q*’) representing mating according to mutual mating fitness [23,24,25,39].

## 5. Information and Non-Random Mating

Sexual selection, defined as any type of selection arising from differential fitness in regard to access to gametes for fertilization [40], can essentially be driven by two biological processes involving non-random mating: competition for mates and mate choice ([25] and the references therein).

In previous work, which has already been mentioned, Carvajal-Rodríguez proposed a simple and abstract framework to describe mate competition and mate choice, along with their consequences in terms of sexual selection and assortative mating. This model is based on the concept of mutual and individual mating fitness. The effects of variation in these fitness measures are described in terms of sexual selection and assortative mating, quantified by the increase in information gained compared to random mating (zero information).

In what follows, we will review how the informational interpretation of Price’s equation connects with the information generated by non-random mating.

Let *q*’(*x*) be the frequency of females in mating, previously noted as *p*’_1_ in [23], and *q*′(*y*) the frequency of males in mating (previously noted as *p*’_2_). Similarly, let *q*(*x*) be the population frequency of females (previously *p*_1_) and *q*(*y*) the population frequency of males (previously *p*_2_). The joint distribution of random mating is *q*(*x*,*y*), where *q*(*x*,*y*) = *q*(*x*)*q*(*y*). Then, the joint distribution, *q*’(*x*,*y*), according to mutual mating fitness, will be equal to [23]:

(9) $$q\prime \left(x,y\right)=m_{xy}q\left(x,y\right)$$

Model (9) relates the frequency, *q*’(*x,y*), of mating guided by the mutual fitness, *m_xy_*, for mating between females in class *x* and males in class *y*, with the frequency *q*(*x*,*y*) = *q*(*x*)*q*(*y*) of random mating between those female and males.

For each pair of classes, we can define a feature or property, *z*(*x*,*y*), about those classes, such that Price’s equation for the mean change in *z* caused by the variation in the mutual propensities, *m*, is:

$$\begin{array}{c} \Delta \bar{z_{m}}=cov\left(m,z\right)=\sum q\left(x,y\right)\left(z\left(x,y\right)-\bar{z}\right)\left(m_{xy}-\bar{m}\right)\\ \Delta \bar{z_{m}}=\sum q\left(x,y\right)\left(z\left(x,y\right)-\bar{z}\right)\left(m_{xy}-1\right)\\ \;\;\;\;\;=\sum q\left(x,y\right)\left(z\left(x,y\right)-\bar{z}\right)\left(\frac{q^{\prime \left(x,y\right)}}{q\left(x,y\right)}-1\right)\\ \;\;\;\;\;\;\Delta \bar{z_{m}}=\sum \Delta q_{xy}z\left(x,y\right) \end{array}$$

where Δ*q_xy_* = *q*’(*x*,*y*) − *q*(*x*,*y*) and ΣΔ*q_xy_* = 0.

So, taking *z*(*x*,*y*) = log(*m_xy_*) = log(*q*’(*x*,*y*)/*q*(*x*,*y*)) we get:

(10) $$\Delta \bar{z_{m}}=\sum \Delta q_{xy}z\left(x,y\right)=\sum \Delta q_{xy}log\left(\frac{q\prime \left(x,y\right)}{q\left(x,y\right)}\right)=J\left(Q\prime ,Q\right)\equiv J_{PTI}$$

as obtained in [23].

Moreover, *q*’(*x,y*)/*q*(*x,y*), when calculated based on observed frequencies, coincides with the pair total index (*PTI*) defined in [41]; *J*(*Q*’,*Q*), which has previously been called *J_PTI_*, and here we will maintain that notation, so *J*(*Q*’,*Q*) ≡ *J_PTI_*.

The *J_PTI_* divergence in (10) can be decomposed into the sum of the information associated with sexual selection (*J_PSS_*), plus the information on assortative mating (*J_PSI_*), plus an interaction that arises when both sexual selection and assortative mating are present [23,25]. However, when the covariance within pairs is zero, *J_PTI_* is reduced to the information corresponding to sexual selection, denoted as *J_PSS_* (see below). When the covariance within pairs is not zero, but the mean and variance between mating and the population are the same, *J_PTI_* is reduced to the information corresponding to assortative mating, referred to as *J_PSI_* [25].

### 5.1. Information and Sexual Selection

As we have said, *J_PSS_* information can be obtained from *J_PTI_* when the covariance in the pairing is 0. This information is the sexual selection information, *J_PSS_*, and it compares the frequencies in the pairing *q*’(*x*)*q*’(*y*) with the population frequencies *q*(*x*)*q*(*y*). In [23], it is proven for the discrete case, that *J_PSS_* is, in turn, the sum of the sexual selection information within each sex, *J_PSS_* = *J_S_*_1_ + *J_S_*_2_, and, in [25], the same is proven for the continuous case. Here, we will provide another demonstration for both cases, continuous and discrete, based on the chain rule for relative entropy, *D_KL_* [36].

We know that:

(11) $$J_{PSS}=J\left(r\left(x,y\right),q\left(x,y\right)\right)=D_{KL}\left(r\left(x,y\right)\left|\right|q\left(x,y\right)\right)+D_{KL}\left(q\left(x,y\right)\left|\right|r\left(x,y\right)\right)$$

where $r\left(x,y\right)=q\prime \left(x\right)\times q\prime \left(y\right)$ and $q\left(x,y\right)=q\left(x\right)\times q\left(y\right).$

And considering each sex separately:

$$J_{S1}=D_{KL}\left(q^{\prime \left(x\right)}\left|\right|q\left(x\right)\right)+D_{KL}\left(q\left(x\right)\left|\right|q^{\prime \left(x\right)}\right)\;J_{S2}=D_{KL}\left(q\prime \left(y\right)\left|\right|q\left(y\right)\right)+D_{KL}\left(q\left(y\right)\left|\right|q\prime \left(y\right)\right)$$

We want to show that *J_PSS_* = *J_S_*_1_ + *J_S_*_2_. To do this, let us consider the conditional probabilities:

(12) $$q\left(y|x\right)=q\left(x,y\right)/q\left(x\right)=\frac{q\left(x\right)q\left(y\right)}{q\left(x\right)}=q\left(y\right)$$

$$r\left(y|x\right)=r\left(x,y\right)/q\prime \left(x\right)=\frac{q\prime \left(x\right)q\prime \left(y\right)}{q\prime \left(x\right)}=q\prime \left(y\right)$$

According to the chain rule [36], we know that:

$$D_{KL}\left(r\left(x,y\right)\left|\right|q\left(x,y\right)\right)=D_{KL}\left(q\prime \left(x\right)\left|\right|q\left(x\right)\right)+D_{KL}\left(r\left(y|x\right)\left|\right|q\left(y|x\right)\right)$$

which due to (12) is as follows:

$$D_{KL}\left(r\left(x,y\right)\left|\right|q\left(x,y\right)\right)=D_{KL}\left(q\prime \left(x\right)\left|\right|q\left(x\right)\right)+D_{KL}\left(q\prime \left(y\right)\left|\right|q\left(y\right)\right)$$

Similarly:

$$D_{KL}\left(q\left(x,y\right)\left|\right|r\left(x,y\right)\right)=D_{KL}\left(q\left(x\right)\left|\right|q\prime \left(x\right)\right)+D_{KL}\left(q\left(y\right)\left|\right|q\prime \left(y\right)\right)$$

Therefore, if we add both divergences and group the *x* and *y* components separately, we see that:

$$J_{PSS}=D_{KL}\left(r\left(x,y\right)\left|\right|q\left(x,y\right)\right)+D_{KL}\left(q\left(x,y\right)\left|\right|r\left(x,y\right)\right)=J_{S1}+J_{S2.}$$

#### Sexual Selection in for Normally Distributed Traits

If traits *x* and *y* are normally distributed, such that mating frequencies, *q*’, follow an *N*(*μ*_1_, Σ_1_) distribution, and population frequencies, *q*, follow an *N*(*μ*_2_, Σ_2_) distribution, where Σ*_i_* is the variance–covariance matrix; then assuming that phenotypic changes are gradual [25], the components *J_S_*_1_ and *J_S_*_2_ coincide with the opportunity for sexual selection index [42].

On the other hand, if we consider the case where the variance in mating is equal to the population variance, *σ*_1*xx*_ = *σ*_2_*_xx_* = *σ_xx_* and *σ*_1_*_yy_* = *σ*_2*yy*_ = *σ_yy_*, this means that *J_S_*_1_ and *J_S_*_2_ equal the square of the standardized sexual selection indices for females (*x*) and males (*y*), respectively. Furthermore, in this case, if there is no assortative mating, the relative entropy is symmetrical, so that *D_KL_*(*q*’||*q*) = *D_KL_*(*q*||*q*’), such that *J_PSS_* = 2*D_KL_*(*q*’||*q*). Therefore, when the mean of the trait is the only difference between the mating and the population frequencies, the sum of the squares for both sexes in terms of the standardized sexual selection indices is equal to twice the relative entropy between the mating frequencies and population frequencies.

To demonstrate the above, we first obtain the *J_PSS_*, assuming that both traits are normally distributed. To do so, we note that the relative entropy between the *Q*’ distribution of the expected pairings, based on mutual fitness, and the *Q* distribution of expected pairings by chance is [25]:

(13) $$D_{KL}\left(q\prime |q\right)=\frac{1}{2}\left(log\left(A\right)-2+\frac{\sigma_{1xx}}{\sigma_{2xx}}+\frac{\sigma_{1yy}}{\sigma_{2yy}}+\frac{\left(\mu_{2x}-\mu_{1x}\right)²}{\sigma_{2xx}}+\frac{\left(\mu_{2y}-\mu_{1y}\right)²}{\sigma_{2yy}}\right)$$

where:

$$A=\frac{\sigma_{2xx}\sigma_{2yy}}{\sigma_{1xx}\sigma_{1yy}-\sigma_{1xy}^{2}}$$

where it has been taken into account that the population covariance in the case of random encounters is 0 i.e., *σ*_2*xy*_ = 0.

The other relative entropy is:

$$D_{KL}\left(q|q\prime \right)=\frac{1}{2}\left(log\left(\frac{1}{A}\right)-2+B\right)$$

where:

$$\begin{array}{c} B\\ =\frac{\sigma_{1xx}\sigma_{2yy}+\sigma_{1yy}\sigma_{2xx}}{\sigma_{1xx}\sigma_{1yy}-\sigma_{1xy}^{2}}\\ +\frac{\sigma_{1yy}\left(\mu_{1x}-\mu_{2x}\right)²+\sigma_{1xx}\left(\mu_{1y}-\mu_{2y}\right)²-2\sigma_{1xy}\left(\mu_{1x}-\mu_{2x}\right)\left(\mu_{1y}-\mu_{2y}\right)}{\sigma_{1xx}\sigma_{1yy}-\sigma_{1xy}^{2}} \end{array}$$

We have already seen that if there is no assortative mating, *q*’(*x,y*) = *q*’(*x*)*q*’(*y*) = *r*(*x*,*y*), and the covariance within the mating is zero, *σ*_1*xy*_ = 0, then:

$$D_{KL}\left(q^{\prime }|q\right)=D_{KL}\left(r|q\right)=\frac{1}{2}\left(log\left(A\right)-2+\frac{\sigma_{1xx}}{\sigma_{2xx}}+\frac{\sigma_{1yy}}{\sigma_{2yy}}+\frac{{\left(\mu_{2x}-\mu_{1x}\right)}^{2}}{\sigma_{2xx}}+\frac{{\left(\mu_{2y}-\mu_{1y}\right)}^{2}}{\sigma_{2yy}}\right)$$

(14) $$D_{KL}\left(q|q\prime \right)=D_{KL}\left(q|r\right)=\frac{1}{2}\left(log\left(\frac{1}{A}\right)-2+\frac{\sigma_{2xx}}{\sigma_{1xx}}+\frac{\sigma_{2yy}}{\sigma_{1yy}}+\frac{\left(\mu_{1x}-\mu_{2x}\right)²}{\sigma_{1xx}}+\frac{{\left(\mu_{1y}-\mu_{2y}\right)}^{2}}{\sigma_{1yy}}\right)$$

and:

$$J_{PSS}=D_{KL}\left(r|q\right)+D_{KL}\left(q|r\right)$$

$$J_{PSS}=-1+\frac{1}{2}\left(\frac{\sigma_{1xx}}{\sigma_{2xx}}+\frac{\sigma_{2xx}}{\sigma_{1xx}}\right)+\frac{1}{2}\left(\mu_{1x}-\mu_{2x}\right)²\left(\frac{1}{\sigma_{1xx}}+\frac{1}{\sigma_{2xx}}\right)+$$

(15) $$-1+\frac{1}{2}\left(\frac{\sigma_{1yy}}{\sigma_{2yy}}+\frac{\sigma_{2yy}}{\sigma_{1yy}}\right)+\frac{1}{2}\left(\mu_{1y}-\mu_{2y}\right)²\left(\frac{1}{\sigma_{1yy}}+\frac{1}{\sigma_{2yy}}\right)$$

where we have regrouped the terms with *x* and *y*, so that they correspond to *J_S_*_1_ + *J_S_*_2_.

Now, in the case where *σ*_1*xx*_ = *σ*_2_*_xx_* = *σ_xx_* and *σ*_1_*_yy_* = *σ*_2*y*_*_y_* = *σ_yy_*, we see that by substituting Equation (14) we obtain *D_KL_*(*q*’||*q*) = *D_KL_*(*q*||*q*’) and by substituting Equation (15) we obtain *J_PSS_* = *SSI*^2^_1_ + *SSI*^2^_2_, where *SSI*_1_ and *SSI*_2_ are the standardized sexual selection indices for females and males, respectively, and, therefore, we get:

$$J_{PSS}={{SSI}^{2}}_{1}+{{SSI}^{2}}_{2}=2D_{KL}\left(q\prime \left|\right|q\right)$$

That is, the information on sexual selection, when there are no covariances and the variances do not change in regard to the mating with respect to the population, is the sum of the square of the standardized selection indices, which in turn coincides with twice the relative entropy between the mating frequencies and the population frequencies.

### 5.2. Information and Assortative Mating

The *J_PTI_* divergence, when we ignore differences between the mating and population frequencies and focus only on frequencies within mating, becomes the *J_PSI_* divergence, which compares, within mating, the distribution due to mutual mating fitness with random mating:

$$J_{PSI}=J\left(q\prime \left(x,y\right),r\left(x,y\right)\right)$$

In previous work, *J_PSI_* has been solved for both the discrete case [23] and the continuous case [25], based on the concept of mutual mating fitness, along with the informational interpretation of Price’s equation. Here, for the continuous case and assuming normal distribution applies, we will derive *J_PSI_* directly from the sum of the relative entropy values when the mean and variance between the mating sample and the population are equal and we will see that it corresponds to a function of the square of the correlation ρ, which is interesting because *ρ* is typically used to detect assortative mating, but here we will show that the information corresponding to that pattern is actually captured by *ρ*^2^/(1 − *ρ*^2^).

As before, we assume that *x* and *y* are normally distributed, such that *q*’ is *N*(*μ*_1_, Σ_1_) and *q N*(*μ*_2_, Σ_2_), where Σ*_i_* is the variance–covariance matrix. As we have seen, the *J_PSI_* divergence focuses only on the mating and the comparison within mating involves two groups of equal means and variances for the trait, so we assume *μ*_1_ = *μ*_2_, *σ*_1*xx*_ = *σ*_2_*_xx_* = *σ_xx_* and *σ*_1*yy*_ = *σ*_2*yy*_ = *σ_yy_*, so what will vary is the covariance within mating, *σ*_1*xy*_.

Then, from Equation (13) we get:

$$D_{KL}\left(q\prime |q\right)=\frac{1}{2}log\left(A\right)$$

and the other divergence:

$$D_{KL}\left(q|q^{\prime }\right)=\frac{1}{2}\left(log\left(\frac{1}{A}\right)-2+\frac{2\sigma_{xx}\sigma_{yy}}{\sigma_{xx}\sigma_{yy}-\sigma_{1xy}^{2}}\right)=\frac{1}{2}log\left(\frac{1}{A}\right)-1+\frac{\sigma_{xx}\sigma_{yy}}{\sigma_{xx}\sigma_{yy}-\sigma_{1xy}^{2}}\;J_{PSI}=D_{KL}\left(q\prime |q\right)+D_{KL}\left(q|q\prime \right)$$

(16) $$J_{PSI}=-1+\frac{\sigma_{xx}\sigma_{yy}}{\sigma_{xx}\sigma_{yy}-\sigma_{1xy}^{2}}=\frac{\sigma_{1xy}^{2}}{\sigma_{xx}\sigma_{yy}-\sigma_{1xy}^{2}}=\frac{\rho ²}{1-\rho ²}$$

We see that assuming normal distribution in regard to the mating, the assortative mating information is a function of the squared correlation coefficient [43].

## 6. Information and Evolution of Gene Frequency in Terms of a General Mating and Fitness Scheme

We have seen that the flow of information generated by the deviation from random mating is calculated by comparing the expected distribution, according to mating fitness, with the expected distribution of random mating. This information is obtained in the form of *J_PTI_* divergence, regardless of whether the distribution is discrete or continuous, based on which appropriate statistics are generated for each case [23,25]. However, model (9), according to which we derived *J_PTI_* in (10), describes phenotypic frequencies (or classes in an infinitesimal interval in the continuous case) without making any assumptions about the underlying genetic model, or connecting it with the biological fitness of the offspring from those instances of mating.

The aim of this section is precisely to connect the description of the mating distribution with the description of the evolution of gene frequencies subject to natural selection and to quantify all of this in terms of information. That is, to generate a model of gene frequency change for any explicit mating model and multilocus diploid genetic system and describe it in terms of information. This remains a pending task for classical population genetics and speciation models [44]; although, recently, information-based models have been developed that transcend the limitations of previous ones. See, for example, [20,22]. These studies use *D_KL_* as a metric that captures the information due to natural selection. However, the distributions these authors use are different; for instance, Smith calculates the information associated with the trajectories by jointly considering the transitions and their states. In any case, the choice of *D_KL_* is ad hoc in the sense that it is defined a priori to compare distributions and does not arise naturally from a model of change due to selection, as *J* divergence does in the case of [18], or in the case of deviations from random mating and sexual selection [23,25].

In what follows, I will propose a model that explicitly incorporates the mating system (the interindividual mating interaction) and that implicitly incorporates the genomic context (mutation, linkage, etc.) into the classical replicator system. From this model, we will see that the mean change in the log fitness caused by the change, at different stages, in the distributions in terms of the mating system, genotypes, and haplotypes, can be captured as information in the form of Jeffrey’s divergence.

We will solve the model for the simple case of one locus: (1) with random mating, thereby recovering the classical replicator system; and (2) with non-random mating, but neutral in terms of viability, thus recovering the mating fitness model defined in [23]. In the latter case, we will use as an example a simple one-locus hybrid sterility model to compare the information on the different Jeffrey’s divergences obtained.

### 6.1. General Model

Let us consider a haplotype *H* (Table 1) of a known frequency in generation *t*, *f*(*H*)*_t_*, as well as the frequencies of the corresponding diploid genotypes, *HH*, *Hh,* and *hh,* where *h* represents any haplotype other than *H*. Likewise the joint distribution *q*’(*x*,*y*), according to the mutual mating fitness for classes *HH*, *Hh,* and *hh,* is given by (9):

*q*’(*x*,*y*) = *m_xy_q*(*x*,*y*)

where *x* represents any female belonging to any of the three classes and *y* represents a male. Then, the general equation for the frequency of *H* in the next generation is:

(17) $$f{\left(H\right)}_{t+1}=\sum_{x,y}q’\left(x,y\right)\times \Phi_{x}\times \left(\omega_{GHH}\pi_{1Hxy}+\sum_{h}\frac{\pi_{2Hhxy}\omega_{GHh}}{2}\right)$$

Equation (17) connects the haplotype frequency *f*(*H*) in the next generation with the distribution of the mating *q*’(*x*,*y*), the fecundity *Φ_x_* of female *x*, and the *G* genotypes of the offspring, namely the carriers of the haplotype. Of these genotypes a proportion, *π*_1_, will be homozygous and, *π*_2_, heterozygous. The genotypes will survive based on their relative fitness *ω* (viability) after the action of natural selection.

The equation is general in the sense that the haplotype component, *H*, incorporates any multilocus genetic system and the *q*’ component can incorporate any mating scheme. However, it is true that the values of π will depend on any mutations or linkage disequilibrium, which in turn depends on the recombination frequency. Therefore, the model will not be dynamically sufficient unless we specify functions for the proportions *π*, which will depend on the case being studied. In each case, we will obtain new statistics for detecting sexual selection and assortative mating, as well as new insights into the underlying evolutionary dynamics.

If there are only two types of haplotypes, the above formula can be simplified as:

$$f{\left(H\right)}_{t+1}=f{\left(HH\right)}_{t+1}+\frac{f{\left(Hh\right)}_{t+1}}{2}=\sum_{x,y}q’\left(x,y\right)\times \Phi_{x}\times \left(\omega_{GHH}\pi_{1Hxy}+\omega_{GHh}\frac{\pi_{2Hxy}}{2}\right)$$

We will now apply (17) to the simple case of a biallelic locus without mutation.

### 6.2. One Locus Model

Let us consider a biallelic locus that codes for a discrete trait with three phenotypes, which are directly associated with their corresponding genotypes and follow a mating scheme described by the mutual mating fitnesses, *m_xy_*. Within these mating fitnesses, we also include the fecundity component *Φ*. We wish to determine the frequency *f*(*A*) of the allele *A* after mating, and after fecundity and viability selection. The frequency *f*(*A*) after selection will be the sum of the frequency of the new *AA* genotypes, plus half of the *Aa* genotypes. The structure of the model is as follows:

(18) $$f{\left(A\right)}_{t+1}=p\prime =f{\left(AA\right)}_{t+1}+\frac{f{\left(Aa\right)}_{t+1}}{2}=\sum_{xy}q{’}_{xy}\times \left(\omega_{AA}\pi_{1Axy}+\omega_{Aa}\frac{\pi_{2Axy}}{2}\right)$$

For convenience, we have denoted *q*’(*x*,*y*) as *q*’*_xy_*, which therefore corresponds to Equation (9). Moreover, *π*_1_*_Axy_* is the fraction of offspring from the mating *x* × *y* that are homozygous for the allele *A*, *π*_2_*_Axy_* is the fraction of offspring from the mating *x* × *y* that are heterozygous for the allele *A*, *ω_AA_* is the relative viability (divided by the average fitness) associated with the homozygote *AA*, and *ω_Aa_* is the relative viability associated with the heterozygote.

To simplify the notation concerning pair parameters, we will use the subscript 1 for the *AA* genotype, 2 for the heterozygote, and 3 for *aa*. Thus, the genotype frequencies in the population before mating are *p*_1_ (*AA* frequency), *p*_2_ (*Aa* frequency), and *p*_3_ (*aa* frequency). If mating is random and we assume that there is no selection in regard to fecundity, we take values *m_xy_* = 1 for all *x*, *y*; the proportion of *AA* and *Aa* genotypes in the offspring before selection and without mutations are shown in Table 2.

If we assume that random mating is taking place, according to (9) we know that the probability of mating between *x* and *y* is *q*’*_xy_* = *q*(*x*,*y*) = *p_x_p_y_* and, noting the allele frequencies of the previous generation denoted as *p* and *q* (without subscripts), we get *p*_1_ = *p*^2^, *p*_2_ = 2*pq,* and *p*_3_ = *q*^2^. Therefore, substituting the values from Table 2 into Equation (18) gets:

$$\begin{array}{c} p\prime =\left[\left(p^{4}+2p³q+p²q²\right)\left(1-\mu_{1\text{\_}}\right)+\mu_{\text{\_}1}f_{1}\left(p,q\right)\right]\omega_{AA}\\ \;\;\;\;\;\;+\frac{1}{2}\left[\left(2p³q+4p²q²+2pq³\right)\left(1-\mu_{2\text{\_}}\right)+\mu_{\text{\_}2}f_{2}\left(p,q\right)\right]\omega_{Aa} \end{array}$$

where *μ*__1_ and *μ*__2_ are any mutations producing genotype *AA* or *Aa*, respectively, *μ*_1__ is any mutation from *AA* to any other genotype and *μ*_2__ from Aa to any other genotype, and *f*_1_ and *f*_2_ are functions of allele frequencies.

Assuming there is no mutation, *μ*_,_ = 0, and after simplifying, we obtain:

$$p\prime =p²\omega_{AA}+pq\omega_{Aa}=p\left(p\omega_{AA}+q\omega_{Aa}\right)={pW}_{A}$$

where *W_A_* = *pω_AA_* + *qω_Aa_* is the marginal fitness of allele *A* [45].

We see that, for a biallelic locus and with random mating, Equations (17) and (18) correspond to the basic model of selection for a biallelic locus [45,46].

#### Genotypic Mating Fitness

Alternatively, if we assume a neutral model, such that *ω_AA_* = *ω_Aa_* = *ω_aa_* = 1, Equation (18) becomes

$$p\prime =p²\left[m_{11}p²+m_{12}2pq+m_{13}q²\right]+pq\left[m_{12}p²+m_{22}2pq+m_{23}q²\right]$$

where we have assumed that the mutual mating fitnesses between classes *x* and *y* do not depend on the sex of the genotype, that is, *m_xy_* = *m_yx_*, and that the genotypic frequencies are equal in males and females.

In these conditions, the individual mating fitness (Formula (4) in [24]) for type *AA* females is:

$$m_{FemAA}=m_{11}p_{1}+m_{12}p_{2}+m_{13}p_{3}$$

and for males:

$$m_{MaleAA}=m_{11}p_{1}+m_{21}p_{2}+m_{31}p_{3}$$

And, since *m_xy_* = *m_yx_*, we get *m_FemAA_* = *m_MaleAA_* = *m_AA_*, thus:

$$m_{AA}=m_{11}p_{1}+m_{12}p_{2}+m_{13}p_{3}$$

In this context, where each phenotypic class corresponds to a genotype, the individual mating fitness *m_AA_* corresponds to the genotypic mating fitness for the *AA* genotype. Then, the frequency of the *AA* genotype in regard to mating is:

(19) $$p\prime_{f1}=p\prime_{m1}=p_{1}m_{AA}=p^{2}m_{AA}$$

Similarly, for heterozygotes, the genotypic mating fitness *m_Aa_* is:

$$m_{Aa}=m_{21}p_{1}+m_{22}p_{2}+m_{23}p_{3}$$

being the frequency of *Aa* in regard to mating:

$$p\prime_{f2}=p\prime_{m2}=p^{2}m_{Aa}=2{pqm}_{Aa}$$

Therefore, using a non-random mating selectively neutral model, the equation for the new frequency of allele *A* in the next generation can be rewritten as:

(20) $$p\prime =p\prime_{1}+\frac{p\prime_{2}}{2}=p²m_{AA}+{pqm}_{Aa}={pM}_{A}$$

where *M_A_* is the marginal mating fitness of allele *A*.

The frequency of allele *A* in the new generation is the sum of the frequencies of genotype *AA*, plus half of the frequency of the heterozygous genotype *Aa* after mating selection. Note that if the genotypic mating fitnesses are 1, meaning that mating is random, then *p*’ = *p*, as expected.

Similarly, for the genotype *aa*, we get:

$$m_{aa}=m_{31}p_{1}+m_{32}p_{2}+m_{33}p_{3}$$

Then, *p*’_3_ = *q*^2^*m_aa_*, and the frequency of allele *a* in the next generation is:

(21) $$q\prime =q²m_{aa}+{pqm}_{Aa}={qM}_{a}$$

where *M_a_* is the marginal mating fitness of allele *a*.

Finally, the change in gene frequency due to differential mating fitness is:

(22) $$\Delta p=p\prime -p=p²\left(m_{AA}-1\right)+pq\left(m_{Aa}-1\right)=p\left(M_{A}-1\right)$$

$$\Delta q=q\prime -q=q²\left(m_{aa}-1\right)+pq\left(m_{Aa}-1\right)=q\left(M_{a}-1\right)$$

The sum of frequencies *p*’ + *q*’ = *p*’_1_ + *p*’_2_ + *p*’_3_ must equal 1. To check this, remember that the mutual mating fitnesses *m_xy_* = *m*’*_xy_*/*M* are normalized (Table 2) and, therefore, the genotypic fitnesses are also *m_AA_* = *m*’*_AA_*/*M*, *m_A__a_* = *m*’*_A__a_*/*M,* and *m_aa_* = *m*’*_aa_*/*M*, where the tilde is used to indicate the unnormalized fitness. The mean fitness, *M* = Σ*_xy_ p_x_p_y_m*’*_xy_*, can be expressed in terms of the unnormalized genotypic fitnesses as:

$$M=p_{1}m{’}_{AA}+p_{2}m\prime_{Aa}+p_{3}m\prime_{aa}=p²m{’}_{AA}+2pqm\prime_{Aa}+q²m\prime_{aa}$$

Therefore using (19)–(21), the sum *p*’ + *q*’ = (*p*^2^*m*’*_AA_* + 2*pqm*’*_Aa_* + *q*^2^*m*’*_aa_*)/*M* = *M*/*M* = 1.

### 6.3. Information and Fitness

Equation (17) can be expressed in terms of diploid genotype frequencies and combined fitness by substituting it with Equation (9), *q*’*_xy_* = *m_xy_q_xy_*, where we obtain:

$$f{\left(H\right)}_{t+1}=\sum_{x,y}q\left(x,y\right)m_{xy}\Phi_{x}\left(\omega_{GHH}\pi_{1Hxy}+\sum_{h}\frac{\pi_{2Hhxy}\omega_{GHh}}{2}\right)$$

Therefore, since the haplotype frequency is simply the sum of the frequencies of the genotypes containing that haplotype, we can also express the frequency of any genotype *G* as:

(23) $$f{\left(G\right)}_{t+1}=\sum_{x,y}q\left(x,y\right)m_{xy}\Phi_{x}\pi_{Gxy}\omega_{G}=\sum_{x,y}q\left(x,y\right)\pi_{Gxy}\Omega_{xyG}$$

where *Ω_xyG_* = *m*_xy_*Φ_x_ω_G_*.

The genotype frequencies in the previous generation can be expressed as:

$$f{\left(G\right)}_{t}=\sum_{x,y}q\left(x,y\right)\pi_{Gxy}$$

For example, if *G* = *AA,* then *f*(*AA*)*_t_* is the sum of the expected frequency according to the chance of crosses *AA* × *AA*, *Aa* × *Aa*, *AA* × *Aa*, and *Aa* × *AA*, and the corresponding proportion of the *AA* genotype in the offspring of each cross, i.e.:

$$\begin{array}{c} f{\left(AA\right)}_{t}=p⁴+\frac{4p²q²}{4}+\frac{2p³q}{2}+\frac{2p³q}{2}=p2\left(p²+q²+2pq\right)=p² \end{array}$$

Note that, in general, the overall fitness component of *G*, *Ω_G_*, is different from the frequency ratio before and after selection:

$$\Omega_{G}\neq \frac{f{\left(G\right)}_{t+1}}{f{\left(G\right)}_{t}}$$

because for each mating, we have *Ω_xyG_* = *m_xy_Φ_x_ω_G_* that can differ for different matings *x*, *y*.

The only fitness element common to genotype *G* will be viability *ω_G_*, so if we assume that there are no differences in the mating fitness or fertility, we get:

$$f{\left(G\right)}_{t+1}=\omega_{G}\sum_{x,y}q\left(x,y\right)\pi_{Gxy}$$

so:

$$\omega_{G}=\frac{f{\left(G\right)}_{t+1}}{f{\left(G\right)}_{t}}$$

And, in this latter case, the mean change in the log fitness caused by selection, due to differential viability, log(*ω_G_*), is:

(24) $$J_{\omega G}=\sum_{x,y,G}\left(f{\left(G\right)}_{t+1}-f{\left(G\right)}_{t}\right)log\left(\omega_{G}\right)$$

#### 6.3.1. Information on Mating by Genotype

However, we are interested in the information due to changes in genotypic frequencies when the different fitnesses causing that change may vary. The problem is that the final frequency of each genotype, which is due to the different mating fitness of its parents, the fecundity of the mother, and ultimately the viability of the genotype itself, cannot be expressed as the result of a single measure of fitness.

One option is to obtain the information associated with each genotype separately. That is, the average change in the logarithm of the overall genotypic fitness, *Ω_xyG_*, after mating (*x*, *y*), associated with the change in frequency of that genotype from one generation to the next, is:

$$\begin{array}{c} J_{G}=\sum_{x,y}\left(f{\left(G\right)}_{\left(t+1\right)xy}-f{\left(G\right)}_{txy}\right)log\left(\Omega_{xyG}\right)\\ \;\;\;\;\;\;=\sum_{x,y}\left(q\left(x,y\right)\pi_{Gxy}\Omega_{xyG}-q\left(x,y\right)\pi_{Gxy}\right)log\left(\Omega_{xyG}\right) \end{array}$$

where it is noted that:

$$f{\left(G\right)}_{txy}=q\left(x,y\right)\pi_{Gxy}$$

Therefore, for the entire set of possible genotypes *G*, whose frequency changes from one generation to the next due to the effect of selection, we get:

(25) $$\Delta G_{\Omega }=\sum_{G}J_{G}$$

That is, if the combined effect of mating fitness, fecundity, and viability produces a shift in genotype frequencies, this can be captured as the sum of the applicable Jeffrey’s divergences.

#### 6.3.2. Genotype Distribution Information

Alternatively, and from Equation (23), we can directly consider the frequency of a genotype as:

$$f{\left(G\right)}_{t+1}=\sum_{x,y}q\left(x,y\right)\pi_{Gxy}\Omega_{xyG}=f{\left(G\right)}_{t}\times \sum_{x,y}f_{xy}\left(\gamma \right)\pi_{Gxy}\Omega_{xyG}\;=f{\left(G\right)}_{t}\times \Omega_{G}$$

where:

$$f_{xy}\left(\gamma \right)\Pi_{Gxy}$$

is a function of the genotype frequencies after the (*x*, *y*) mating and *Ω_G_* is the overall marginal fitness of genotype *G*.

$$\Omega_{G}=\sum_{x,y}f_{xy}\left(\gamma \right)\pi_{Gxy}\Omega_{xyG}=\frac{f{\left(G\right)}_{t+1}}{f{\left(G\right)}_{t}}$$

So, the mean change in the overall log fitness caused by the variation in genotype frequencies because of selection takes the form of Jeffrey’s divergence, as follows:

(26) $$J_{\Omega G}=\sum_{\gamma }\left(f{\left(\gamma \right)}_{t+1}-f{\left(\gamma \right)}_{t}\right)log\left(\Omega_{\gamma }\right)$$

#### 6.3.3. Haplotype Distribution Information

From Equation (17), we can directly consider the frequency of haplotype *H* as:

$$f{\left(H\right)}_{t+1}=\sum_{x,y}q\left(x,y\right)\left(\Omega_{xyGHH}\pi_{1Hxy}+\sum_{\eta \neq H}\frac{\Omega_{xyGH\eta }\pi_{2H\eta xy}}{2}\right)=\;f{\left(H\right)}_{t}\left(g_{1}\left(f\left(H\right)\right)\Omega_{xyGHH}\pi_{1Hxy}+\sum_{\eta \neq H}g_{2}\left(f\left(\eta \right)\right)\frac{\Omega_{xyGH\eta }\pi_{2H\eta xy}}{2}\right)$$

where *η* is any allele other than *H*, and *g*_1_ and *g*_2_ are functions of the frequency in the previous generation of *H* and *η*, respectively.

Then:

$$f{\left(H\right)}_{t+1}=f{\left(H\right)}_{t}\Omega_{H}$$

where:

$$\Omega_{H}=g_{1}\left(f\left(H\right)\right)\Omega_{xyGHH}\pi_{1Hxy}+\sum_{\eta \neq H}g_{2}\left(f\left(\eta \right)\right)\frac{\Omega_{xyGH\eta }\pi_{2H\eta xy}}{2}$$

is the marginal overall fitness of haplotype *H*.

So, the total mean change in the log fitness caused by the change in haplotype frequencies is:

(27) $$J_{\Omega \eta }=\sum_{\eta }\left(f{\left(\eta \right)}_{t+1}-f{\left(\eta \right)}_{t}\right)log\left(\Omega_{\eta }\right)$$

Therefore, from model (17), if we measure the mean change in the log fitness, we can obtain different measures of information associated with the change in genotype frequency or the change in haplotype frequency. These types of information are generally different, since there can be genotypic change without a change in gene frequency. As for the change in genotypes, we calculate the information in two ways, one linked to the variation in each genotype obtained from different instances of mating and another linked to the variation in the genotype frequencies as a whole in the new population.

Below are some simple examples, in this regard, for a biallelic locus.

#### 6.3.4. Sexual Selection Information for a Biallelic Locus

We will assume, like before, that the genotypic frequencies in males and females are equal; in the case of females, we already saw that, *p*’*_f_*_1_ = *p_f_*_1_*m_AA_*, *p_f_*_1_ = *p*^2^, *p*’*_f_*_2_ = *p_f_*_2_*m_Aa_*, *p_f_*_2_ = 2*pq*, etc. The difference between the population frequencies, *p_f_*, and the frequencies within the mating, *p*’*_f_*, is *Δp_f_*_1_ = *p*’*_f_*_1_ − *p_f_*_1_ = (*p*_1_)*m_AA_* − (*p*_1_), etc.

Information due to sexual selection in females in terms of genotypic mating fitness can be described as:

$$J_{S1}=\sum \Delta p_{fx}log\frac{p\prime_{fx}}{p_{fx}}\;J_{S1}=\left[\left(p_{1}m_{AA}-p_{1}\right)logm_{AA}+\left(p_{2}m_{Aa}-p_{2}\right)logm_{Aa}+\left(p_{3}m_{aa}-p_{3}\right)logm_{aa}\right]$$

and since the genotypic frequencies in males and females are equal, *p_fx_* = *p_my_*, and we have assumed that the mutual fitnesses are symmetrical, *m_xy_* = *m_yx_*, then, sexual selection in males is equal to that of females, *J_S_*_2_ = *J_S_*_1_, and, since *J_PSS_* = *J_S_*_2_ + *J_S_*_1_, then:

(28) $$J_{PSS}=2\left[p²\left(m_{AA}-1\right)log\left(m_{AA}\right)+2pq\left(m_{Aa}-1\right)log\left(m_{Aa}\right)\;+q²\left(m_{aa}-1\right)log\left(m_{aa}\right)\right]$$

where we can see that Jeffreys’ divergence for sexual selection captures the information generated by the change in genotype frequencies, weighted by the logarithm of the genotypic mating fitness.

We can now compare, for this same scenario, what the genotypic information (Equations (25) and (26) and the haplotypic information (Equation (27)) would be.

Equation (25) computes the information generated by the change in the distribution of genotypes as a sum of the information:

$$\Delta G_{\Omega }=\sum_{G}J_{G}$$

where:

$$J_{G}=\sum_{x,y}\left(f{\left(G\right)}_{\left(t+1\right)xy}-f{\left(G\right)}_{txy}\right)log\left(\Omega_{xyG}\right)$$

In our case, this implies that *m_xy_* = *m_yx_* and that *Ω_xy__G_* = *m_xyG_*, then:

$$J_{AA}=\left(p^{4m_{11}}-p^{4}\right)log\left(m_{11}\right)+2\left(p^{3}qm_{12}-p^{3}q\right)log\left(m_{12}\right)\;+\left(p^{2}q^{2m_{22}}-p^{2}q^{2}\right)log\left(m_{22}\right)\;p²\left(p^{2m_{11}}-p^{2}\right)log\left(m_{11}\right)+p²\left(2pqm_{12}-2pq\right)log\left(m_{12}\right)\;+p²\left(q^{2m_{22}}-q^{2}\right)log\left(m_{22}\right)\;p²\left[p²\left(m_{11}-1\right)log\left(m_{11}\right)+2pq\left(m_{12}-1\right)log\left(m_{12}\right)\;+q²\left(m_{22}-1\right)log\left(m_{22}\right)\right]$$

Similarly:

$$J_{aa}=q²\left[q²\left(m_{33}-1\right)log\left(m_{33}\right)+2pq\left(m_{32}-1\right)log\left(m_{32}\right)\;+p²\left(m_{22}-1\right)log\left(m_{22}\right)\right]$$

and, finally:

$$\begin{array}{c} J_{Aa}=2pq[p²\left(m_{12}-1\right)log\left(m_{12}\right)+pq\left(m_{22}-1\right)log\left(m_{22}\right)\\ \;\;\;\;\;\;+q²\left(m_{23}-1\right)log\left(m_{23}\right)+pq\left(m_{13}-1\right)log\left(m_{13}\right)] \end{array}$$

Now recall that:

$$m_{AA}=p^{2m_{11}}+2{pqm}_{12}+q^{2m_{13}}\;m_{Aa}=p^{2m_{21}}+2{pqm}_{22}+q^{2m_{23}}\;m_{aa}=p²m_{31}+2{pqm}_{32}+q²m_{33}$$

We can see that the difference between *J_PSS_* and *J_G_* is where we focus the unit of information. That is, for the sexual selection information, *J_PSS_*, the focus is on each genotype that is involved in various instances of mating. So, for *m_AA_*, the focus is on the combination of mating that involves one or two *AA* genotypes as parents. However, for *J_G_* information, the focus is within a specific genotype produced by the mating.

As for the other measure of information that we have defined for the genotypes (Equation (26)):

$$J_{\Omega \eta }=\sum_{\eta }\left(f{\left(\eta \right)}_{t+1}-f{\left(\eta \right)}_{t}\right)log\left(\Omega_{\eta }\right)\;f{\left(G\right)}_{t+1}=\sum_{x,y}q\left(x,y\right)\pi_{Gxy}\Omega_{xyG}=f{\left(G\right)}_{t}\times \sum_{x,y}f_{xy}\left(\gamma \right)\pi_{Gxy}\Omega_{xyG}=f{\left(G\right)}_{t}\times \Omega_{G}$$

which in regard to our sexual selection scenario implies:

$$f{\left(AA\right)}_{t+1}=p^{2\left(p^{2m_{11}}+2pqm_{12}+q^{2m_{22}}\right)}\;f{\left(AA\right)}_{t}=p^{2\left(p^{2}+2pq+q^{2}\right)}=p^{2}\;\Omega_{AA}=p²m_{11}+2pqm_{12}+q²m_{22}\;f{\left(Aa\right)}_{t+1}=2pq\left[p^{2m_{12}}+pq\left(m_{22}+m_{13}\right)+q^{2m_{23}}\right]\;f{\left(Aa\right)}_{t}=2pq\;\Omega_{Aa}=p^{2m_{12}}+pq\left(m_{22}+m_{13}\right)+q^{2m_{23}}\;f{\left(aa\right)}_{t+1}=q^{2\left[q^{2m_{33}}+2{pqm}_{32}+p^{2m_{22}}\right]}\;f{\left(aa\right)}_{t}=q^{2}\;\Omega_{aa}=q²m_{33}+2{pqm}_{32}+p²m_{22}$$

so that:

$$J_{\Omega \eta }=p²\left(\Omega_{AA}-1\right)log\left(\Omega_{AA}\right)+2pq\left(\Omega_{Aa}-1\right)log\left(\Omega_{Aa}\right)\;+q²\left(\Omega_{aa}-1\right)log\left(\Omega_{aa}\right)$$

We can see that the difference between *J_PSS_* in Equation (28) and *J_Ω__η_* is due to the different fitness weights of each genotype and, since *m_xy_* = *m_yx_*, when comparing these aspects, we can see that:

$$\Omega_{AA}-m_{AA}=q^{2\left(m_{22}-m_{13}\right)}\;\Omega_{Aa}-m_{Aa}=pq\left(m_{13}-m_{22}\right)\;\Omega_{aa}-m_{aa}=p²\left(m_{22}-m_{13}\right)$$

The difference lies in the crosses, *Aa* × *Aa* (*m*_22_) and *AA* × *aa* (*m*_13_ or *m*_31_). This is because the *J_PSS_* calculation of sexual selection focuses on the variation generated by the mating on the marginal frequency of each genotype. For example, the marginal frequency of *AA* comes from the mating of *AA* with other genotypes, which includes the cross *AA* × *aa* (mating fitness *m*_13_), but not *Aa* × *Aa* (mating fitness *m*_22_). The same applies to the marginal frequency of the aa genotype; it includes the cross *aa* × *AA,* but not the cross between heterozygotes.

On the other hand, the calculation of the genotypic information, *J_Ω__η_*, directly studies the variation in the frequency of each genotype resulting from a set of mating. In this case, the pattern is reversed, namely for the genotypes *AA* and *aa*, the cross between heterozygotes is included, but not the *AA* × *aa*. The opposite holds true for the *Aa* genotype.

In this specific case involving a biallelic locus with no differences in terms of viability and fertility, but with mating fitness under the constraint *m_xy_* = *m_yx_*, if the mating fitness among heterozygotes coincides with the mating fitness among different homozygotes, the information obtained from the genotypes aligns with the information from sexual selection. If this condition is not met, the information is similar, but differs by a factor associated with the difference between *m*_22_ − *m*_13_.

Finally, for the haplotype information measure (Equation (27)), the information generated by the change in the distribution of haplotypes is:

$$J_{\Omega \eta }=\sum_{\eta }\left(f{\left(\eta \right)}_{t+1}-f{\left(\eta \right)}_{t}\right)log\left(\Omega_{\eta }\right)$$

where:

$$f{\left(\eta \right)}_{t+1}=f{\left(\eta \right)}_{t}\Omega_{\eta }\;\Omega_{\eta }=g_{1}\left(f\left(\eta \right)\right)\Omega_{xyG\eta \eta }\pi_{1\eta xy}+\sum_{h\neq \eta }g_{2}\left(f\left(h\right)\right)\frac{\Omega_{xyG\eta h}\pi_{2\eta xy}}{2}$$

which in our case implies:

$$\begin{array}{c} f{\left(A\right)}_{t+1}=p[\left(p³m_{11}+2p²qm_{12}+pq²m_{22}\right)\\ \;\;\;\;\;\;+\left(p²{qm}_{12}+pq²\left(m_{22}+m_{13}\right)+q³m_{23}\right)] \end{array}$$

so:

$$\Omega_{A}=p³m_{11}+3p²qm_{12}+pq²\left(2m_{22}+m_{13}\right)+q³m_{23}$$

and:

$$\begin{array}{c} f{\left(a\right)}_{t+1}=q[\left(q³m_{33}+2pq²m_{32}+pq²m_{22}\right)\\ \;\;\;\;\;\;+\left(p²{qm}_{12}+pq²\left(m_{22}+m_{13}\right)+q³m_{23}\right)] \end{array}$$

so:

$$\Omega_{a}=q³m_{33}+3pq²m_{32}+p²q\left(2m_{22}+m_{13}\right)+p³m_{12}$$

Information about the change in allele frequency caused by selection is defined as:

$$J_{\Omega \eta }=p\left(\Omega_{A}-1\right)log\left(\Omega_{A}\right)+q\left(\Omega_{a}-1\right)log\left(\Omega_{a}\right)$$

Note that if the mating fitnesses, *m_ij_*, are equal to one, then *Ω_A_* = *Ω_a_* = 1 and, therefore, the information is zero.

#### 6.3.5. Toy Example: Information for Hybrid Sterility

Let us compare the different information indices we have defined using a simple hybrid sterility model. We continue with the one-locus model without mutations and assume that heterozygotes have lower fertility, so that their frequency in terms of successful mating (with fertilization) is lower. That is, *m*’_12_ = *m*’_21_ = *m*’_22_ = *m*’_23_ = *m*’_32_ = *ε* < 1. The rest of the mating fitnesses equal one, *m*’_11_ = *m*’_13_ = *m*’_31_ = *m*’_33_ = 1. The average mating fitness is:

$$M=\left(p²+q²\right)m\prime_{\varepsilon }+2pq\varepsilon$$

where:

$$m\prime_{\varepsilon }=p²+2pq\varepsilon +q²$$

Since the frequencies of males and females are equal and *m*’*_ij_*= *m*’*_ji_*, then the marginal fitnesses of females and males are equal:

$$m\prime_{AA}=m\prime_{aa}=m\prime_{\varepsilon }\;m\prime_{Aa}=\varepsilon$$

The normalized frequencies will be:

$$m_{11}=m_{13}=m_{31}=m_{33}=\frac{1}{M}=m\;m_{12}=m_{21}=m_{22}=m_{23}=m_{32}=\frac{\varepsilon }{M}=Ε\;m_{AA}=\frac{m\prime_{AA}}{N}=m_{aa}=m\prime_{aa}=m_{\varepsilon }=\frac{m\prime_{\varepsilon }}{M}\;m_{Aa}=Ε$$

##### Sexual Selection Information

Substituting (28) we obtain:

$$J_{PSS}=2\left[\left(p²+q²\right)\left(m_{\varepsilon }-1\right)log\left(m_{\varepsilon }\right)+2pq\left(Ε-1\right)log\left(Ε\right)\right]$$

*J_PSS_* represents the information obtained from the change in the phenotypic frequencies of each sex (in this case, equal to the genotypic frequencies) within the mating, with respect to the population frequencies.

Note that in the particular case of equal allelic frequency, *p* = *q*, we get:

$$m\prime_{\varepsilon }=\frac{1+\varepsilon }{2}\;M=\frac{1+3\varepsilon }{4}\;m_{\varepsilon }=\frac{2\left(1+\varepsilon \right)}{1+3\varepsilon }\;Ε=\frac{4\varepsilon }{1+3\varepsilon }$$

and the value of *J_PSS_* is not 0:

$$J_{PSS}=\frac{\left(1-\varepsilon \right)}{1+3\varepsilon }log\left(\frac{\left(1+\varepsilon \right)}{2\varepsilon }\right)$$

We can see that if, for example, we take *ε* = 1/3, we obtain *J_PSS_* = (1/3)log(2) ≠ 0. This is interesting because this model with equal allele frequencies does not affect the distribution of genotypes in the following generation and, therefore, the information obtained from the distributions of genotypes and haplotypes (alleles) will be 0 (see below).

##### Information on Mating by Genotype

We can calculate the sum of the *J_G_* indices, which for each genotype compute the frequency of the genotype after the mating with respect to the expected frequency, if the instances of mating are random. This mating information on each genotype corresponds to Equation (25) and, in our example, is as follows:

$$J_{AA}=p^{2}\left[p²\left(m-1\right)log\left(m\right)+\left(1-p^{2}\right)\left(Ε-1\right)log\left(Ε\right)\right]\;J_{Aa}=2pq\left[pq\left(m-1\right)log\left(m\right)+\left(1-pq\right)\left(Ε-1\right)log\left(Ε\right)\right]\;J_{aa}=q²\left[q²\left(m-1\right)log\left(m\right)+\left(1-q²\right)\left(Ε-1\right)log\left(Ε\right)\right]$$

The total change for the three genotypes will be the result of the following sum:

$$\Delta G_{\Omega }=J_{AA}+J_{Aa}+J_{aa}$$

Note that in the case *p* = *q*, *J_AA_* = *J_aa_* = *J*, *J_Aa_* = 2*J*. Then, Δ*G_Ω_* = 4*J*.

$$J=\frac{-3}{16}\frac{\left(1-\varepsilon \right)}{\left(1+3\varepsilon \right)}log\left(\varepsilon \right)\;\Delta G_{\Omega }=\frac{-3}{4}\frac{\left(1-\varepsilon \right)}{\left(1+3\varepsilon \right)}log\left(\varepsilon \right)$$

taking *ε* = 1/3, Δ*G_Ω_* = (1/4)log(3) ≠ 0.

We see that the mating information by genotype is not 0 either, even though the allele frequencies are equal. This is because the distribution of genotypes according to mating changes, even though the total distribution remains constant.

##### Genotype Distribution Information

We can use Equation (26) to calculate the information obtained when the distribution of genotypes changes. In this case, the fitness associated with each genotype is:

$$\Omega_{AA}=p^{2}m+\left(1-p^{2}\right)Ε\;\Omega_{Aa}=pqm+\left(1-pq\right)Ε\;\Omega_{aa}=q²m+\left(1-q²\right)Ε$$

And the information is:

$$J_{G\Omega }=p²\left(\Omega_{AA}-1\right)log\left(\Omega_{AA}\right)+2pq\left(\Omega_{Aa}-1\right)log\left(\Omega_{Aa}\right)\;+q²\left(\Omega_{aa}-1\right)log\left(\Omega_{aa}\right)$$

Note that if *p* = *q* = 0.5, the three values of *Ω_G_* are equal and *m* = 4/(1 + 3*ε*) and *E* = 4*ε*/(1 + 3*ε*), so that

$$\Omega_{G}=\left(\frac{1}{4}\right)m+\left(\frac{3}{4}\right)E=\frac{1}{\left(1+3\varepsilon \right)}+\frac{3\varepsilon }{1+3\varepsilon }=1$$

and the information is *J_ΩG_* = 0.

This is expected, since selection acts on heterozygotes and the gain in frequency in terms of homozygotes is distributed equally (in the case of *p* = *q*), generating heterozygotes again and, therefore, the genotypic distribution is not altered when the frequencies of both alleles are equal.

##### Haplotype Distribution Information

We calculate this information using Equation (27) and, as such, we obtain:

$$\Omega_{A}=p\Omega_{AA}+q\Omega_{aa}\;\Omega_{a}=q\Omega_{aa}+q\Omega_{aa}$$

and the corresponding information is:

$$J_{\eta }=p\left(\Omega_{A}-1\right)log\left(\Omega_{A}\right)+q\left(\Omega_{a}-1\right)log\left(\Omega_{a}\right)$$

which, just like in the previous case, will be 0 if *p* = *q*.

## 7. Discussion

In this work, we have proposed a population genetics model that begins with a mating system guided by mutual mating fitness between individuals, continues with the distribution of genotypes resulting from each mating, and follows with the subsequent survival of these genotypes, based on their viability fitness until they reach adulthood. At each stage of the model, variation in the distribution of the genotypes is captured in terms of information.

Population genetics models are fundamental both for testing proposed hypotheses, whether general or associated with a specific species and evolutionary scenario, and for deepening our understanding of the history of species and biodiversity on Earth. They also help reveal invariances and logical connections that, in turn, may suggest new questions and pose new hypotheses [47,48].

As mentioned in the introduction, information theory seems to be increasingly relevant in terms of providing new descriptions of the evolutionary process, allowing us to explore certain aspects more deeply or facilitate the development of models and estimators for parameters of interest. Recent studies have shown how selection accumulates information in the genome [20] and also how the flow of information throughout the process of evolutionary change can capture different degrees of adaptation and can help develop new statistics [22].

It should be noted that when we consider typical equations in terms of classical population genetics, or the replicator equation as *p*’ = *Ωp*, where *Ω* is some kind of relative fitness, and we express the mean change in the logarithm of the fitness of the character, what we obtain is an equation of the type:

$$\Delta \bar{z_{s}}=\sum_{i}p_{i}f\left(\Omega_{i}\right)$$

where *f* is the generating function (*Ω* − 1)log(*Ω*) of an *f* divergence corresponding to Jeffrey’s divergence.

Therefore, every component of the evolutionary process that is guided by some kind of selection, be it sexual selection, mate choice, or viability, etc., such that we can express the expected change in terms of (*Ω* − 1)log(*Ω*), can be described in terms of Jeffrey’s divergence. That is, the dynamics of evolutionary change itself, at least as it can be expressed in the given form *p*’ = *pΩ*, can be measured in terms of Jeffrey’s divergence, because that is what it is mathematically, in this sense it is not an ad hoc statistic.

In the model presented here, the average change in fitness at different stages of the life cycle can be calculated as Jeffrey’s information. This has allowed us to calculate the information associated with various stages of the process, namely the information obtained from the distribution of mating, the information associated with mating for each genotype, as well as the information associated with the distribution of genotypes and haplotypes in the new generation. We have found that these indices contain distinct information and, therefore, could allow us to draw inferences about associated phenomena.

Population genetics models typically assume random mating [20,47] or, at most, consider any mating system implicitly, as in [21,22], where different mating systems may be reflected in the topology of a graph. In our case, by explicitly modeling the mating system in terms of information, on the one hand, we unify different aspects related to the detection of sexual selection and assortative mating and facilitate the development of statistical measures [25]. And, on the other hand, by incorporating mating fitness into a more general model, we reveal connections between different mating processes and other parts of the gene frequency evolution process, described in terms of information.

An interesting aspect of the proposed model is that the average changes in terms of different types of fitness, due to variations between the corresponding distributions, can be easily translated into statistical tests, since Jeffrey’s divergence approximates well a chi-square distribution, when the hypothesis of equality between the distributions is met [36,49].

All this has allowed the development of a multi-model selection methodology applied to the estimation of non-random mating [24] that can be used in empirical studies, see for example [50,51,52].

### Limitations of the Model

The proposed model connects the mating in the parental generation with the distribution of adult genotypes in the next generation. The model explicitly accounts for the mating system defined by mutual mating fitness, female fecundity, and the viability associated with genomic genotypes. The effects of mutation and recombination are not explicitly modeled, as they are implicitly included in the calculation of genotypic proportions following mating. However, in its current form, the model does not consider migration, genetic drift, epigenetic inheritance, or other potential factors related to niche constructions or habitat choice [53].

However, these aspects are not central to the main message of the work, which emphasizes the connection between different aspects of evolutionary change and the measurement of information through shifts in distributions, a point also highlighted in recent studies [20,22].

## 8. Conclusions

In conclusion, it may be worth considering the relationship between biological fitness and information, whether the increase in information is favored by selection [54], or even whether we can think of fitness in terms of multilevel information alignment between populations and their environment [55,56]. Additionally, we might reflect on the advantages of shifting from a fitness-driven approach to evolution toward one guided by information and whether this could help clarify, broaden, and deepen the mathematical description of evolutionary theory.

## Figures and Tables

**Table 1:** A table of main symbols used in Section 6.

Symbol	Meaning
*q*	Mating frequency if mating is random.
*m_xy_*	Relative mutual mating fitness between female with phenotype *x* and male with phenotype *y*, i.e., the propensity of mating with fertilization between a given pair of phenotypic classes.
*q*′	Mating frequency after the action of mutual mating fitness.
*Φ_G_*	Fecundity, which is included within mutual mating fitness.
*M*	Average mutual mating fitness.
*m_G_*	The relative (divided by the average *M*) marginal mating fitness of genotype *G*.
*ω_G_*	The relative (divided by the average fitness *W*) genotype viability, i.e., the potential for survival to adulthood of a given genotype.
*Ω_xyG_*	The product of *m_xy_ Φ_G_ ω_G_*.
*H*	A given haplotype, i.e., a sequence that carries a particular set of genes that tend to be inherited together.
*Π*_1*Hxy*_	A fraction of the offspring from the mating (*x*, *y*) that are homozygous for the *H* haplotype.
*Π*_2*Hxy*_	A fraction of the offspring from the mating (*x*, *y*) that are heterozygous for the *H* haplotype.
*p*_1_, *p*_2_, *p*_3_	For one locus, frequencies of the genotypes *AA*, *Aa*, and *aa*, respectively. These symbols are used to simplify the notation of mating frequencies in regard to single-locus examples.

**Table 2:** Proportion *p_x_p_y_π* of offspring genotypes *AA* and *Aa* as a function of mating frequencies, when *m_xy_* = 1 for every *x*, *y*.

Mating	Offspring	
	*π*_1*A*_ (*AA*)	*π*_2*A*_ (*Aa*)
*p*_1_ × *p*_1_ × *m*’_11_/*M*	*p*_1_^2^	0
*p*_1_ × *p*_2_ × *m*’_12_/*M*	(*p*_1_*p*_2_)/2	(*p*_1_*p*_2_)/2
*p*_1_ × *p*_3_ × *m*’_13_/*M*	0	*p*_1_ *p*_3_
*p*_2_ × *p*_1_ × *m*’_21_/*M*	(*p*_1_*p*_2_)/2	(*p*_1_*p*_2_)/2
*p*_2_ × *p*_2_ × *m*’_22_/*M*	(*p*_2_^2^)/4	(*p*_2_^2^)/2
*p*_2_ × *p*_3_ × *m*’_23_/*M*	0	(*p*_2_*p*_3_)/2
*p*_3_ × *p*_1_ × *m*’_31_/*M*	0	*p*_1_ *p*_3_
*p*_3_ × *p*_2_ × *m*’_32_/*M*	0	(*p*_2_*p*_3_)/2
*p*_3_ × *p*_3_ × *m*’_33_/*M*	0	0
*M* = Σ*_xy_ p_x_p_y_m*’*_xy_*		

Note that *m_xy_* = *m*’*_xy_*/*M*.

## Data Availability

Data are contained within the article.
